# Identification, Biochemical Characterization, and Safety Attributes of Locally Isolated *Lactobacillus fermentum* from *Bubalus bubalis* (buffalo) Milk as a Probiotic

**DOI:** 10.3390/microorganisms10050954

**Published:** 2022-04-30

**Authors:** Sana Abid, Arshad Farid, Rameesha Abid, Mujeeb Ur Rehman, Walaa F. Alsanie, Majid Alhomrani, Abdulhakeem S. Alamri, Syed Mohammed Basheeruddin Asdaq, Daniel Ingo Hefft, Saddam Saqib, Muhammad Muzammal, Sabrin Abdelrahman Morshedy, Mashael W. Alruways, Shakira Ghazanfar

**Affiliations:** 1 Department of Biology, Faculty of Science & Technology, Virtual University, Lahore 54000, Pakistan; sanaabid933@gmail.com; 2 Gomal Centre of Biochemistry and Biotechnology, Gomal University, D.I.Khan 29050, Pakistan; arshadfarid@gu.edu.pk (A.F.); mustafamuzammal1@yahoo.com (M.M.); 3 Department of Biotechnology, University of Sialkot, Sialkot 51310, Pakistan; rameesha.abid@uskt.edu.pk; 4 National Institute of Genomics and Advanced Biotechnology (NIGAB), National Agricultural Research Centre, Park Road, Islamabad 45500, Pakistan; 5 Department of Pharmacy, Quaid-i-Azam University, Islamabad 45500, Pakistan; mujeebkhwaja@gmail.com; 6 Department of Clinical Laboratory Sciences, The Faculty of Applied Medical Sciences, Taif University, Al Hawiyah 21944, Saudi Arabia; w.alsanie@tu.edu.sa (W.F.A.); m.alhomrani@tu.edu.sa (M.A.); a.alamri@tu.edu.sa (A.S.A.); 7 Centre of Biomedical Sciences Research (CBSR), Deanship of Scientific Research, Taif University, Al Hawiyah 21944, Saudi Arabia; 8 Department of Pharmacy Practice, College of Pharmacy, AlMaarefa University, Dariyah 13713, Saudi Arabia; sasdag@mcst.edu.sa; 9 Reaseheath College, University Centre Reaseheath, Nantwich CW5 6DF, UK; daniel.hefft@reaseheath.ac.uk; 10 Department of Biotechnology, Mohi- ud-Din Islamic University, Nerian Sharif 12080, Pakistan; saddamsaqib.qau@gmail.com; 11 Fish and Animal Production Department, Faculty of Agriculture (Saba Basha), Alexandria University, Alexandria 21526, Egypt; sabrin_morshedy@alexu.edu.eg; 12 Department of Clinical Laboratory Sciences, College of Applied Medical Sciences, Shaqra University, Shaqra 15273, Saudi Arabia; m.alruways@su.edu.sa

**Keywords:** fermented milk, identification, *Lactobacillus fermentum*, probiotics, safety attributes

## Abstract

The demand of functional foods is on the rise, and researchers are trying to develop nutritious dairy products by using well-characterized strains of bacteria. In this study, we identified locally isolated strains of *Lactobacillus fermentum* from *Bubalus bubalis* (Nilli Ravi buffalo) milk and evaluated their potential as probiotics in food products like fermented milk. Fifteen *Lactobacillus* strains were initially isolated, and only four strains (NMCC-2, NMCC-14, NMCC-17, and NMCC-27) were examined for morphological and biochemical characterizations due to their ability of gas production in Durham tubes. Moreover, these strains were selected for further probiotic characterizations due to their extreme morphological resemblance with lactic acid bacteria for their antimicrobial activity, enzymatic potential, autoaggregation capability, hydrophobicity, and acid and bile tolerance. All selected isolates showed significant probiotic potential. However, NMCC-14 and NMCC-17 strains showed maximum probiotic potential. The isolates (NMCC-2, NMCC-14, NMCC-17, and NMCC-27) were identified as *Lactobacillus fermentum* utilizing 16S rRNA gene sequencing. The in vivo safety study of NMCC-14 (dose: 10^10^ CFU/day/mice; 21 days, orally) showed no histological dysfunctions in a mouse model. Pathogenic bacterial enzymes reduced the beneficial bacterial load in the host gastrointestinal tract. These results suggest that the NMCC-14 strain is safe and can be potentially used as a probiotic. Moreover, fermented milk was prepared by using the NMCC-14 strain. The results revealed that NMCC-14 strain-based fermented milk had significantly (*p* < 0.05) higher protein content (4.4 ± 0.06), water-holding capacity (WHC), and dynamic viscosity as compared to non-fermented milk. The results suggest that L. *fermentum* NMCC-14 is safe and nontoxic; hence, it can be a beneficial supplement to be used for the development of dairy products to be subjected to further clinical testing.

## 1. Introduction

Probiotics are live bacteria and yeasts that are used for their beneficial properties in both animals and humans. They are often called helpful bacteria, important for the digestive system because of their capability to contribute to gut health [1,2]. They were discovered in the early 20th century and received prime consideration in the 21st century [3,4]. The idea of using microbes to process food is not new and was already applied to produce cheese and fermented products by Greeks and Romans in ancient times. The Russian scientist Elie Metchnikoff (often considered the father of probiotics) observed and speculated that health and gut microbiome can be improved by host-friendly bacteria while using stale or acerbic milk [5]. Since then, the study of probiotics has continued, and interest has been increasing, because of their importance and applications, within the scientific community as well as food and pharmaceutical industries [6,7]. The term probiotic comes from the ancient Greek word “pro” meaning ”for” and “biotic” meaning “life” [8]. Studies have revealed that *Lactobacilli* are constituents of the intestinal microflora of animals and humans [9]. Several probiotics such as *Enterococci, Bifidobacterium, Leuconostoc* spp. and *Saccharomyces* spp. were tested and are utilized to improve food quality and human nutrition [10]. However, *Lactobacillus* is the most common beneficial class of probiotics for various organisms [11] and is present in fermented dairy products such as yogurt, kefir, and buttermilk [12,13]. Due to the wide use of probiotics in fermentation, *Lactobacillus* is also added to other foods such as pickled vegetables, kimchi, pao cai, miso, and soy sauce [14,15,16]. Species of this genus are also famous for producing lactic acid and are commonly called Lactic Acid Bacteria (LAB) [17]. The genus *Lactobacillus* represents a hefty, assorted cluster of Gram-positive, non-spore-forming anaerobic bacteria [18]. Microbiologists and food biotechnologists associate LAB with health-improving properties for animals, and for this said reason, scientists frequently exploit LAB in developing dietary foods for animals [19,20]. Furthermore, LAB-incorporating products are especially used in infant foods, various types of processed milk, and pharmaceutical and nutraceutical products [21,22]. In 2006, the World Health Organization (WHO) approved *Lactobacillus* and *Bifidobacterium* species as good probiotics which are safe for human consumption [23,24]. It is evident from previous studies that when probiotics are used in food, they enhance the immune system response in the host, help in digestion, and modulate the gastrointestinal (GIT) microbiota [19,25]. Furthermore, probiotics are also helpful in the treatment of different disorders such as gastrointestinal disorders, immune diseases, and inflammatory bowel disease [26,27,28]. The use of antibiotics can be reduced upon consumption of probiotics because probiotics play a very crucial role in improving human health [29,30]. Hence, it is essential to explore the beneficial microbiota and to expand the applications of bacteria as probiotics in the food and pharmaceutical industries. However, little work has been conducted related to the identification, isolation, and use of probiotic *Lactobacillus* bacteria from animals’ gut [31]. In this study, we hypothesized that buffalo milk possesses a large diversity of *Lactobacillus* probiotics that would be interesting to examine and identify. We isolated and identified *Lactobacillus* strains based on 16S rRNA from buffalo milk. Buffalo (*Bubalus bubalis*) is a commercially important animal in different regions of Asia; hence, there is potential to make use of this animal’s *Lactobacillus* strains to improve public health [32]. Moreover, this research also characterized the probiotic potential of the isolated strains and evaluated their safety profile through a mouse model.

## 2. Material and Methodology

### 2.1. Milk Sample Collection from Buffalos

Fresh milk samples were collected from lactating dairy buffalos (*n* = 30) using sterile gloves within a period of two months (June and July 2018) in the Livestock Research Station (LRS), National Agricultural Centre (NARC), Islamabad, Pakistan. Animal research was carried out in accordance with the ARRIVE guidelines [33]. Milk was collected in the early morning, stored in sterile containers (Deltalab, Spain), and transported to the Microbial Biotechnology Laboratory, NARC. The samples were then stored at −20 °C for bacterial strains isolation.

### 2.2. Isolation of Bacterial Strains from Buffalo Milk

MRS (De Man, Rogosa Sharpe Agar, Himedia) medium was used for the isolation and purification of bacteria from the milk samples [34]. The milk samples were spread on MRS plates by the serial dilution method using sterile distilled water. Serial dilutions were made up to 5 folds. Then, 100 μL of each serially diluted sample was poured into already prepared MRS medium and incubated at 37 °C for 48 h. The purification of the colonies was carried out by using the steak plate method. Morphological characterization, including color, shape, margin, elevation, texture, and size, was determined by following Bergey’s manual [35]. Different biochemical tests were performed, such as Gram staining, evaluation of catalase, oxidase, urease, methyl red, citrate, triple sugar iron, gas formation, and fermentation for the identification of lactic acid bacteria isolates. The results were interpreted according to “Bergey’s Manual of Determinative Bacteriology” [36]. Gram staining was performed and evaluated by phase-contrast microscope (Phase contrast 2, Nikon, Tokyo, Japan).

### 2.3. Determination of Probiotic Potential

#### 2.3.1. Bile and Acid Resistance

The acid tolerance of the isolates was assessed by following the approach of Yu and Zhang [37]. In short, samples were centrifuged and suspended in saline (pH 2.0, 3.0, and 7.0) for the experiment. The bile salt tolerance was assessed by following the technique of Vinderola and Reinheimer [38]. MRS-THIO broth supplemented or not with 0.5% and 1% ox gall (*w/v*) of bile (Sigma Aldrich, Burlington, MA, USA) was used for the bile salt resistance test. Acid and bile resistance was determined by the plate count method.

#### 2.3.2. Lysozyme, Pepsin, and Pancreatin Tolerance

Lysozyme tolerance potential was assessed by using the technique described by Zhang and Liu [39]. In detail, lysozyme and pancreatin along with pepsin were mixed in phosphate-buffered saline (PBS) with an 18 h- fresh culture and centrifuged. After centrifugation, the pellets were washed with PBS and incubated with lysozymes (0.2 mg/mL, pH 8.0, and 1 h incubation), pepsin (0.3 mg/mL, pH 2.0 and 3 h incubation) and pancreatin (1 mg/mL, pH 8.0, and 5 h incubation) to check the potential of the isolated bacteria. Resistance was calculated in percentage [40].

#### 2.3.3. Autoaggregation and Hydrophobicity Evaluation

Autoaggregation was evaluated in all isolates by following the approach of Collado and Meriluoto [41]. Bacterial cultures were centrifuged after 18 h, and the pellets were washed with PBS. The pellets were resuspended in PBS and incubated at 37 °C for 5 h. We took aliquots of 0.2 mL of supernatant after 0, 2, and 5 h intervals and measured their OD (optical density) at 600 nm. The hydrophobicity assay was performed by following the approach of Collado et al. [41]. The isolated bacterial cells were centrifuged and subsequently washed with PBS. Each pellet was resuspended in PBS. P-xylene was added to the suspensions and gently mixed. The medium was incubated at 37 °C for 20 min, then the solvent phase was isolated, and the absorbance was recorded at 600 nm.

### 2.4. Enzymatic Activity of Lactobacillus

#### 2.4.1. Bile Salt Hydrolase Activity

To assess bile salt hydrolase (BSH) activity, a sterile cork was punched in Petri plates to obtain 6 mm-diameter wells in MRS medium, in which we placed 0.5% bile salt, cholic acid, and deoxycholic acid (Sigma Aldrich, Burlington, MA, USA). Subsequently, 200 µL of MRS broth inoculated with an overnight culture of *Lactobacillus* spp. was added to each well. The appearance of a white precipitate in the plates confirmed BSH activity of the isolated strains [42].

#### 2.4.2. Proteolytic Activity

To assess the proteolytic potential of the isolated strains, colorimetric Azo groups released from artificial azocasein were quantified (Sigma Aldrich, Burlington, MA, USA). For this, 50 µL of each *Lactobacillus* spp. was mixed with 500 µL of azocasein and incubated at 37 °C for 2 h. To terminate the reaction, 500 µL of 10% tricarboxylic acid (TCA) was added. The mixture was centrifuged at 1300 rpm for 12 min. A part of the supernatant was mixed with 50% NaOH. The absorbance of the Azo groups was examined at 450 nm, whereas the proteolytic activity was quantified by total enzymatic yield (absorbance: 440 nm = 0.01 per min).

#### 2.4.3. Amylase Activity

The amylase assay was performed by utilizing 3,5-dinitrosilicylic acid (DNS) following the method described by Siddharth [43]. Moreover, protein concentrations in cell extracts were determined by the Bradford method. The release of 1 µmole of enzyme/mg/min under the reaction conditions was considered as the amylase activity.

### 2.5. Safety Profile

#### 2.5.1. Hemolytic Potential

To determine the hemolytic potential, the isolated strains were inoculated in MRS medium and subsequently streaked on blood agar-enriched medium with sterile 5% defibrinated blood. The medium was incubated at 37 °C for 48 h. Finally, the appearance of deep hemolysis zones was evaluated.

#### 2.5.2. Antibiotic Tolerance

Antibiotic resistance was examined using the disc diffusion method and commercially available antibiotics (streptomycin; Scientific Laboratory Supplies, ciprofloxacin; DAILYMED, vancomycin; HIMEDIA, metronidazole; Accord-UK-Ltd, ampicillin; Cdila Pharma, chloramphenicol; FLINN SCIENTIFIC, kanamycin; Fischer Scientific, erythromycin; FLINN SCIENTIFIC, penicillin; REYOUNG PHARMACEUTICALS; and tetracycline; Fischer Scientific). The isolated strains were inoculated in MRS broth and incubated anaerobically at 37 °C for 24 h. The antibiotics discs were placed on already streaked plates to check the resistance of the strains.

### 2.6. Antimicrobial Activity

The antimicrobial activity of different LAB strains was evaluated by using the well diffusion method [44] against different pathogenic strains such as *E. coli* (ATCC8739); *Pseudomonas aeruginosa* (ATCC9027), *Staphylococcus aureus*; (ATCC6538); *Listeria monocytogenes* (ATCC13932), and *Bacillus cereus* (ATCC-11778). The diameter of the zone of inhibition was measured (mm) at the end of the experiment.

### 2.7. Identification of the Selected Isolates by 16S rRNA Gene Sequencing

For the extraction of template DNA from pure bacterial colonies, a single colony of each strain was picked and mixed with 20 μL of Tris-EDTA buffer in PCR strips. The mixture was heated (95 °C) for 10 min in a PCR machine (Conventional PCR, Thermo Fisher Scientific, Waltham, MA, USA). After centrifugation, the supernatant was removed and served as a template DNA. Amplification of the 16S rRNA gene was performed using the PCR machine. A total of 25 μL TAKARA Pre-mix Ex-Taq; 2 μL of universal forward primer 9F (5′-GAGTTGATCCTGGCTCAG-3′), 2 μL of universal reverse primer 1510R (5′-GGCTA CCTTGTTACGA -3′), 20 μL PCR water, and 1 μL template DNA (total volume 50 μL) were used for the PCR amplification of DNA. The amplified PCR products were sequenced by Macrogen sequencing, Korea (http://dna.macrogen.com, accessed on 10 February 2019). The strains were identified at the species level by using the EzBioCloud server (https://www.ezbiocloud.net/identify, accessed on 10 February 2019).

### 2.8. In Vivo Safety Assessment in a Mouse Model

Albino mice (male, *n* = 20) weighing 23 ± 3 grams (age: 6–8 weeks) were purchased from the National Institute of Health, Islamabad, Pakistan, and housed in the animal cantonment facility of NIGAB, NARC. The animals were first acclimatized for 5 days to the new environment. The research animals were divided into two equal groups (*n* = 10), i.e., a control group without probiotic treatment, receiving a standard basal diet (BD), and a probiotic-treated (*Lactobacillus fermentum* NMCC-14, dose: 10^10^ CFU/day/animal for 21 days) group. All mice experiments were performed according to the “guidelines and principles of laboratory animals” provided by the Bioethical Committee of Quaid-i-Azam University, Islamabad, with approval number BEC-FBS-QAU2021-266.

### 2.9. Histological Examination

Mice were sacrificed by cervical dislocation on the 21st day after 24 h since the last administration of *Lactobacillus fermentum* NMCC-14 (dose: 10^10^ CFU/day/animal, per oral rout). Colon tissue samples were first washed in 0.9% normal saline and then preserved in 10% formalin for histological examination. The samples were then embedded in paraffin and cut into 5 µm-thick sections using a rotary microtome. Hematoxylin-and-eosin (H&E) staining was performed by following these steps: first, wax removal was performed by xylene treatment; the sections were then passed through alcohol to remove xylene and hydrated by thorough rinsing with water. Hematoxylin nuclear staining followed by bluing with a weakly alkaline solution was performed. The sections were then counter-stained with an eosin solution to visualize nonnuclear elements. In the last stage, alcohol treatment and dehydration, followed by xylene rinsing, were performed to clear the tissue and enhance its transparency. The prepared sections were finally mounted with a thin film of polystyrene, and coverslips were applied. The slides were then examined for histological changes using an optical light microscope (Olympus, by Olympus Corporation, Tokyo, Japan).

### 2.10. Cecal Bacterial Count, β-Glucuronidase, and β-Glucosidase tests

Cecal samples were aseptically collected for further analysis of colony-forming unit per gram and the determination of β-glucuronidase and β-glucosidase concentrations by following the method of Sung-Ho et al. [45].

#### Production of Fermented Milk

Milk was inoculated with a 2% bacterial culture and incubated for fermentation (24 h) at 37 °C in closed ampules [46]. After fermentation, the milk was stored at 4 °C for 28 days, and survivability of bacteria, changes in protein contents, lactose, pH, water-holding capability (WHC), syneresis, and dynamic viscosity of the fermented milk were measured after 25 days by using the AOAC techniques 2000.

### 2.11. Ethics Statement

All study protocols were approved by the Ethical and Biosafety Committee of the National Agricultural Research Center (NARC, Islamabad, Pakistan) and performed in accordance with the Ethical and Biosafety Committee of National Agricultural Research Center (NARC, Islamabad: Approval No. IBC-NARC 2020-1 dated 15-12-2020). The animal research was carried out in accordance with the ARRIVE guidelines [33].

### 2.12. Statistical Analysis

Experimental data ware recorded as mean ± standard deviation of triplicates. Furthermore, the data were statistically analyzed through Graph pad prism 5 software. Significant differences were determined through Tukey’s range post ANOVA test set at *p* < 0.05.

## 3. Results

Morphological and biochemical screening of the isolates was performed to assess the viability and stability of the isolates in the presence of essential enzymes, and viable isolates with beneficial probiotic potential were selected for further examination. A total of 92 bacterial colonies were purified on MRS medium from Nilli Ravi buffalo milk. Fifty-five isolates showed typical small pinpointed colonies and were catalase-negative, Gram-positive, and oxidase-negative, typical of LAB. They were randomly selected and preserved in glycerol (35%) for future tests. Out of these 55 bacterial isolates, 15 strains showed maximum cell viability at pH 1–3. The results showed that bacterial isolates remained stable at pH 2 and pH 3 without any substantial decline in viability (Table 1). Probiotic bacterial strains have been observed to tolerate an environment at pH 3 for 2.5 h. Resistance to low pH is an important criterion for the selection of probiotic strains, as unfavorable conditions make it difficult for microorganisms to thrive in the stomach during transit, where food has to be processed for 2–3 h [47].

**Figure 1:**
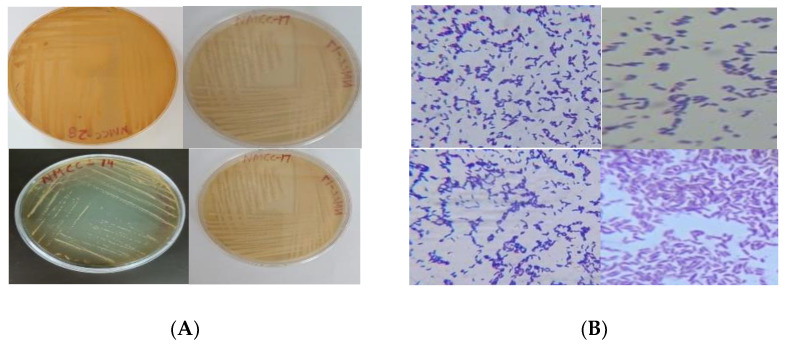
(**A**) Colony morphology; (**B**) Gram staining of the isolated probiotics strains.

The gas production potential of all strains in glucose broth was checked for about 5 days. Only four strains showed gas production in Durham tubes and were selected for further testing (Table 2, Figure 1).

**Figure 2:**
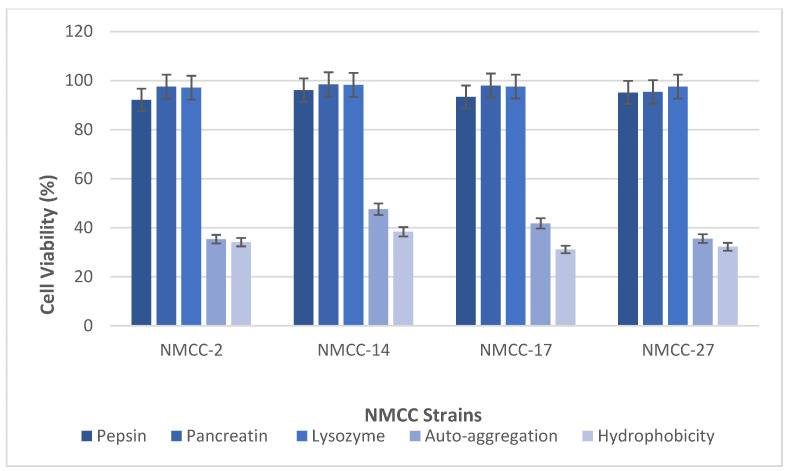
Evaluation of different physiological properties of LAB strains. In vitro values (*n* = 3) are mean ± standard deviations. Different subscripts lowercase letters indicate significant differences (*p* < 0.05).

The effect of enzymes on the isolated species was assessed using pepsin, pancreatin, lysozyme and by analyzing autoaggregation capacity and hydrophobicity. The selected isolates showed significant viability in the presence of pepsin, pancreatin, and lysozyme; NMCC-14 showed the highest viability in the presence of enzymes. Autoaggregation and hydrophobicity indicated adhesion capacity of the selected strains. In this regard NMCC-14 revealed the highest autoaggregation and hydrophobicity values of 47.55 ± 0.08, 82.34 ± 0.04 (Figure 2).

**Figure 3:**
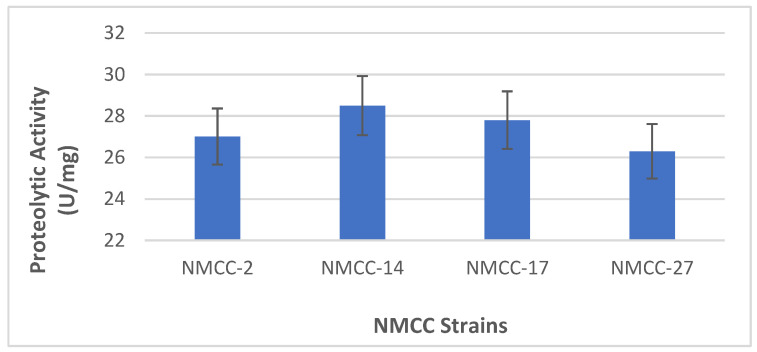
Proteolytic potential of the isolated LAB strains.

**Figure 4:**
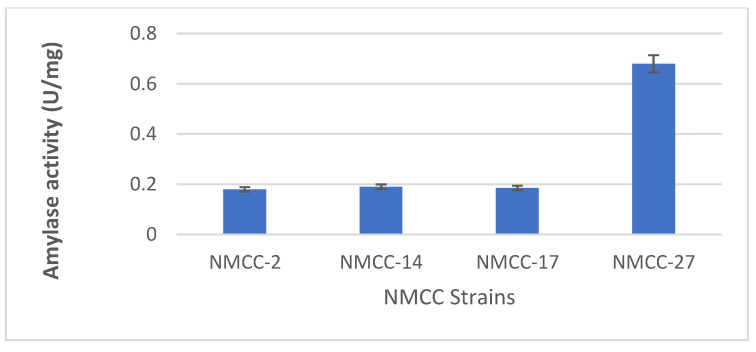
Amylolytic potential of the isolated LAB strains.

**Figure 5:**
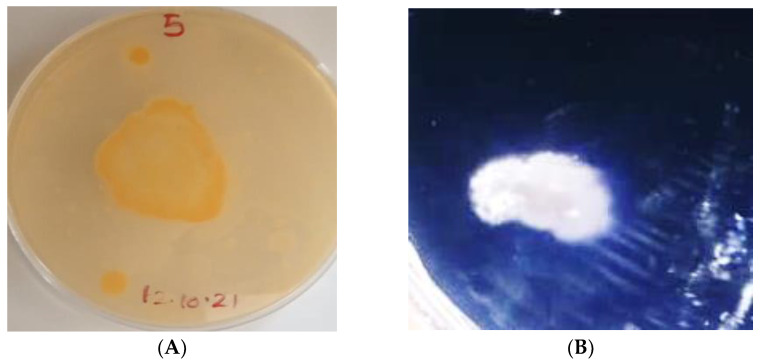
(**A**) Amylolytic potential; (**B**) Proteolytic potential of the isolated LAB strain NMCC-14.

The LAB strains selected in the primary evaluation for further biochemical characterizations revealed a zone of hydrolysis. In quantitative analysis, the protease and amylase activities of NMCC-14 and NMCC-17 were the highest at 28.5 ± 0.1 U/mg and 27.8 ± 0.3 U/mg, respectively (Figure 3, Figure 4 and Figure 5).

**Figure 6:**
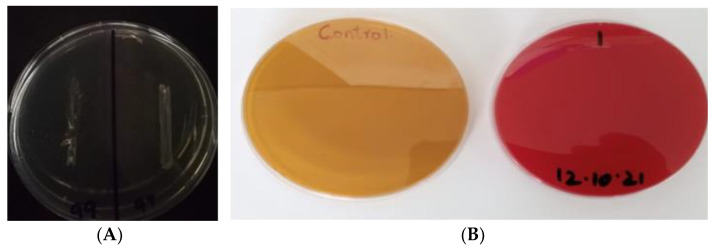
Safety profile: (**A**) DNAs; (**B**) Hemolytic activity. The test plates inoculated with all the isolates studied revealed white zones.

The appearance of white precipitates in the medium revealed that all selected strains produced BSH enzyme. NMCC-17 and NMCC-14 revealed a more prominent white zone in the medium, while, for NMCC-2 and NMCC-27, the white zones were not very prominent, indicating poor BSH enzyme production (Figure 6; Table 3).

**Figure 7:**
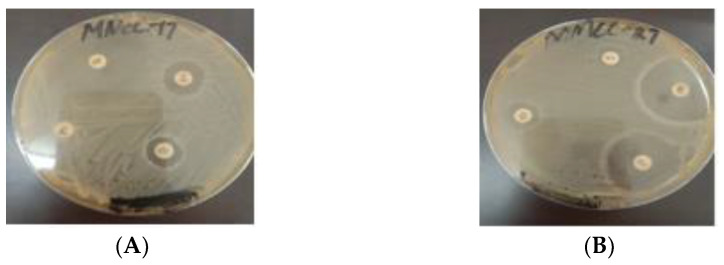
Antibiotic activity: (**A**) NMCC-17; (**B**) NMCC-27.

All tested *Lactobacillus* strains were mostly susceptible to most of the analyzed antibiotics (including erythromycin, clindamycin and ampicillin). Studies have reported that *Lactobacillus* spp. showed resistance against antibiotics such as cefazolin, penicillin, gentamicin, ampicillin, amikacin, and chloramphenicol. The sensitivity of the bacterial isolates against antibiotics is reported in Table 4 and Figure 7.

Various types of pathogenic bacterial species can also be inhibited by probiotic bacteria. In this study, *E. coli* (ATCC8739), *Pseudomonas aeruginosa* (ATCC9027), *Staphylococcus aureus* (ATCC6538), *Listeria monocytogenes* (ATCC13932), and *Bacillus cereus* (ATCC-11778) strains were used as indicator pathogens. Impressively, NMCC-14 exhibited resistance against all pathogenic strains; thus, it could be exploited as a potential antimicrobial probiotic candidate against animal pathogens. The inhibition activity of the bacterial isolates against various food-borne pathogens is reported in Table 5.

**Figure 8:**
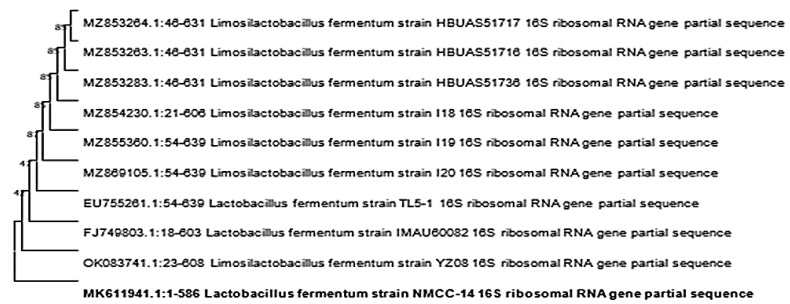
Phylogenetic tree of *Lactobacillus fermentum* NMCC-14 bacterial strain. Bootstrap values can be seen at each node.

**Figure 9:**
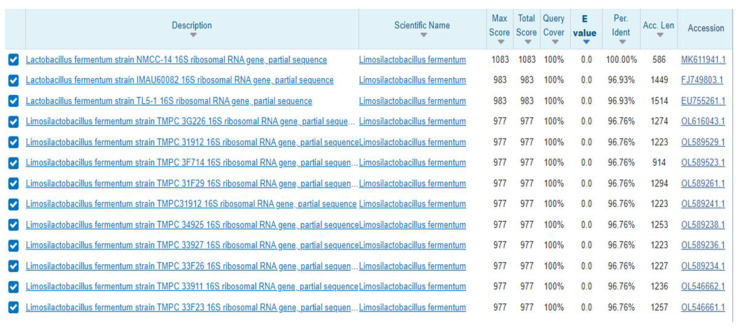
Blast results shows similarity to *Lactobacillus fermentum*.

**Figure 10:**
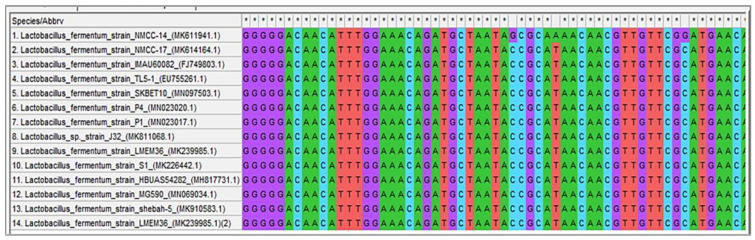
Multiple sequence alignment of *Lactobacillus fermentum* NMCC-14 bacterial strain.

All characterized strains (NMCC-2, NMCC-14, NMCC-17, and NMCC-27) were identified as *Lactobacillus fermentum* strains on the basis of their genotype and showed similarities of 93.66%, 93.79%, 93.41%, and 93.22%, respectively. The results obtained for the NMCC-14 strain are shown in Figure 8. Conservation and homology of the isolated strains were observed by multiple sequence alignment. Along with conserved regions, nucleotide differences were also identified. A phylogenetic tree was constructed based on the unambiguously aligned 16S rRNA gene sequence of three bacterial species identified in the study using MEGA-X (Molecular Evolutionary Genetics Analysis) software. The 16S rRNA sequence of the provisionally identified strain NMCC-14 was submitted to NCBI GenBank under the accession number MK611941. Different color codes are used in Figure 8, Figure 9 and Figure 10 for each nucleotide to show homology and differences in the obtained sequences.

**Figure 11:**
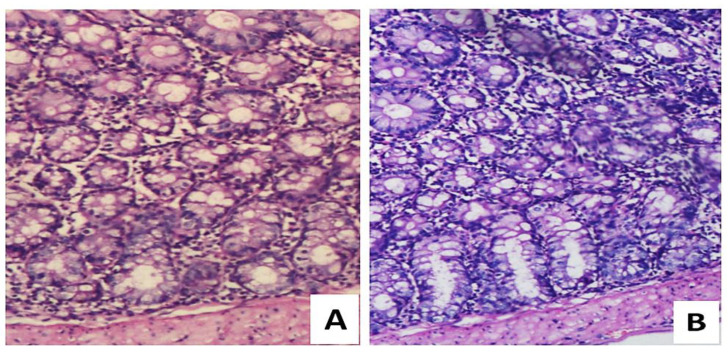
Microscopic study of hematoxylin and eosin (H&E) staining of colon tissue samples. (**A**) Control group; (**B**) *Lactobacillus fermentum* NMCC-14-treated group. Compared to the control, probiotic-treated mice showed no cellular damage in the colon. Integrity in cellular structure was maintained, with no goblet cells depletion and the enhancement of crypt formation.

To evaluate the effect(s) of *Lactobacillus fermentum* NMCC-14, mice were administered the potential probiotic (dose: 10^10^ CFU/day, per oral route), and changes in their colon were investigated in comparison to the colon on the control mice. Histological examination showed that no notable abnormalities or mucosal damage was observed in the colon of *Lactobacillus fermentum* NMCC-14-administered mice (Figure 11). When examining cellular integrity, we observed improvement in goblet cells architecture and enhancement in crypt formation induced by *Lactobacillus fermentum* NMCC-14. The in vivo safety profile of NMCC-14 showed that the oral administration of *Lactobacillus fermentum* NMCC-14 for up to 21 days showed no notable abnormalities or the mucosal damage the mice colon tissues compared to control samples (H&E staining, Figure 11).

**Figure 12:**
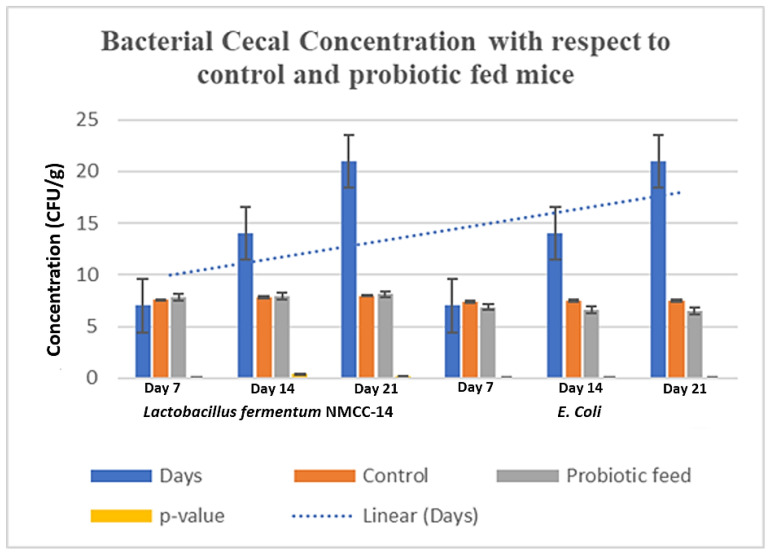
Cecal concentration of *Lactobacillus fermentum* NMCC-14 and *E. coli* in control and probiotic-fed mice.

Cecal bacterial count (CFU/g) showed that the probiotic-administered animals had a significantly (*p* < 0.05) higher *Lactobacillus fermentum* NMCC-14 population as compared to the control group. The concentration of *E. coli* was significantly (*p* < 0.05) decreased in the probiotic-fed mice as compared to the control group (Figure 12).

**Figure 13:**
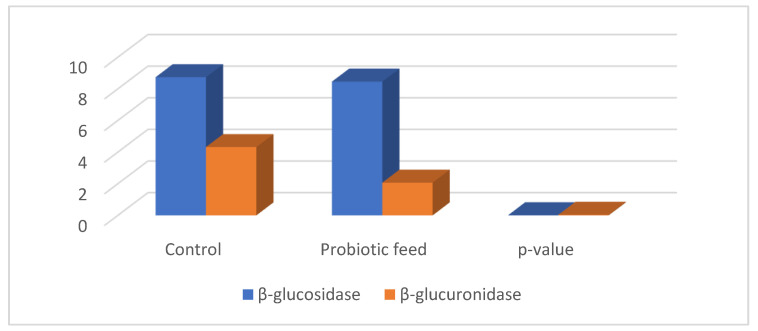
Cecal concentration of β-glucuronidase and β-glucosidase in control and probiotic-fed mice.

The diet supplemented with the probiotic *Lactobacillus fermentum* NMCC-14 significantly lowered (*p* < 0.01) β-glucuronidase and β-glucosidase (*p* > 0.05) concentrations as compared to the control diet (Figure 13).

The fermentation properties of *Lactobacillus fermentum*-NMCC-14 (accession no. MK611941) and NMCC-17 were further analyzed in milk. The protein and lactose contents were compared to those of milk before inoculation of the LAB strains. The concentration of fat remained 0.25% during fermentation (Table 6).

## 4. Discussion

In this study, the isolation and characterizations of LAB strains obtained from buffalo milk is reported. Among them, four strains showed significant biochemical properties and enzymatic potential which were further analyzed. These strains were examined morphologically and revealed significant biochemical potential. In addition, high acidification rate, antimicrobial activity, autoaggregation capacity, hydrophobicity, and biofilm forming potential were observed. To select appropriate bacterial strains for probiotic applications, resistance to low pH is important, as unfavorable conditions (pH 2–3) make it difficult for microorganisms to thrive in the stomach, where food has to be processed for 2–3 h [1]. The survivability of probiotic bacteria is highly strain-dependent [8]. Therefore, in order to confirm the enzymatic potential of the bacterial strains, subsequent characterization of the selected strains was performed via different biochemical analyses. Furthermore, it is well established that acid tolerance is an exclusive property of each strain and varies immensely among LAB species and between strains [11].

The results of this study indicated that the bacterial isolates remained stable at pH 2 and pH 3, without any substantial decline in viability. Previous studies reported that probiotic bacterial strains have been observed to tolerate pH 3 for 2.5 h [48,49]. In this study, pH adjusted at 3 was used to measure the acid tolerance capacity of *Lactobacillus* strains, as this is considered the standard pH to investigate the acid tolerance of probiotic strains in various studies [50]. Several studies have shown tolerance and survival of *Lactobacillus fermentum* at pH 3. This high acid tolerance might be due to ATPase activity. In contrast, it is noteworthy that in some studies *Lactobacillus* spp. showed a severe decline in growth at pH 2.5 and grew only at pH 5, 6, and 7 [48].

LAB exhibit a complex system of proteinases and peptidases that help them to use casein as a source of amino acids and nitrogen [51]. The process involves cell wall-associated proteases, which cleave casein to oligopeptides and help in providing amino acids and nitrogen. Therefore, the high proteolytic activity will help LAB to survive, supporting milk storage. Amylase activity in NMCC-14 and NMCC-17 was also the highest, 0.19 ± 0.03 U/mg and 0.185 ± 0.02 U/mg, respectively, compared to that of other LAB. These results suggest that the action of extracellular and intracellular proteolytic and amylase enzymes contributed to the high proteolytic and amylatic activity. Safety assay results showed that all selected strains (NMCC-2, NMCC-14, NMCC-17, and NMCC-27) had no ability to degrade blood (gamma hemolysis) and DNA [52]. Some studies showed that LAB cells can completely hydrolyze α and β-casein in 4 h of digestion at 30 °C. Śliżewska and Chlebicz-Wójcik [53] indicated that LAB strains showed complete hydrolysis in probiotic-treated yogurt as compared to control. Hence, the results of the current study also indicate that the utilization of LAB bacteria in milk products will help to prolong storage and preservation time.

The isolated bacterial strains showed a varying degree of resistance towards most of the antibiotics that were tested. NMCC-17 showed sensitivity towards chloramphenicol and erythromycin, in accordance with previous research [54,55]. However, in other studies, *Lactobacillus* spp. were reported to be resistant to erythromycin and chloramphenicol [50]. Interestingly, NMCC-17 showed complete resistance to kanamycin, streptomycin, tetracycline, vancomycin, ciprofloxacin, metronidazole, and penicillin, while being intermediately resistant to ampicillin.

The antimicrobial potential of *Lactobacillus* spp. has been reported in numerous studies. *Lactobacillus fermentum* and *Lactobacillus salivarius* were reported to exhibit anti-staphylococcal effects. L. *fermentum* reduced the growth of planktonic *S. aureus*, whereas L. *salivarius* reduced the growth of planktonic *S. aureus*, as well as its biofilm. Proteomic analysis revealed the bactericidal effect of *Lactobacilli* was due to five distinct anti-staphylococcal proteins [56]. Similarly, other studies reported the antagonistic effects of L. *fermentum* towards *E. coli, P. aeruginosa, Salmonella* spp., *Shigella sonnei*, and *some* enterotoxigenic *S. aureus* strains [57].

*Lactobacillus* contains a broad range of antibacterial compounds such as lactic acid, acetic acid, bacteriocins, retrocyclin, and various kinds of peptides [58]. Recently, the use of antimicrobial growth promoters (AGP) in animal feed has increased rapidly. As an alternative to AGP, probiotics are now widely used in animal feed [59]. Particularly, the yeast *Saccharomyces cerevisiae* is used and is mostly is imported. Thus, the isolation and identification of *Lactobacillus* spp. will contribute to the production of antimicrobial agents.

Digestive enzymes such as pepsin, pancreatin, and lysozyme hold much attention since they are involved in a wide range of reactions. Moreover, these enzymes play a significant role in many industrial processes also in the food industry. Therefore, the isolated strains were evaluated against digestive enzymes to determine the probiotic potential of the selected strains. The results of this study are in accordance with another study [60] which showed a high viability of *Lactobacillus* strains in the presence of pepsin, pancreatin, and lysozyme. Results suggested that these strains have a significant ability to survive in the harsh conditions of the gastric and intestinal environment. In the autoaggregation analysis, the isolated strains NMCC-17 and NMCC-27 showed better results in the initial 5 h, with high viability in contrast with a previous study [49],that reported that *Lactobacilli* strains were not viable in the presence of pepsin [49]. The results of viability, autoaggregation ability, and hydrophobicity of our study are in accordance to findings reported in the literature [47]. These results suggested that selected strains may be used as antimicrobial preservatives and food additives in animals and human food. Therefore, both isolates were selected for molecular identification. A phylogenetic tree showed the relationship between the strains NMCC-2, NMCC-14, NMCC-17, and NMCC-27 and already published *Lactobacillus fermentum* strains on the basis of their genotype. *Lactobacillus fermentum* strains were seen clustering as the outgroup of the mega cluster of *fermentum* strains which showed some divergence from other closely related strains. Previous studies conducted in Pakistan revealed that *Lactobacillus* was the most prevalent group in Pakistani yogurt samples, while some strains of *Streptococcus* were also reported. Phylogenetic tools provide significant means to researchers for the identification of unexplored species [4].

*Lactobacillus* strains are recognized as GRAS for their potential use as probiotics [4]. The FAO (Food and Agriculture Organization) strongly recommends that newly isolated and characterized strains be tested for their safety before being used food/feed supplements. Furthermore, the in vivo probiotic potential of the identified strains was evaluated in milk samples, because all biochemical and physiological tests in our study confirmed that both strains were valuable for application in dairy products. Particularly, the results of this study revealed that NMCC-17 showed the highest acidification rate. Furthermore, this strain also showed the lowest syneresis in products (20%), potentially lower than that of classical yogurt. The water-holding capacity (WHC) of the product prepared with NMCC-14 was comparable to that of classical milk. These results suggest that the isolated NMCC-14 strain has potential as a pre-culture or functional component of fermented milk. This study suggests that the described strains can be used for the preparation of functional dairy products.

## 5. Conclusions

The purpose of this study was to isolate and identify comparatively most promising probiotic strains for milk fermentation that can facilitate both qualitatively and quantitatively the manufacturing of functional foods. Fifteen *Lactobacillus* strains were initially isolated and only four strains were examined for morphological and biochemical characterizations due to their ability of gas production in Durham tubes. This study demonstrated that two *Lactobacillus* strains (NMCC-14 and NMCC-17) isolated from buffalo milk showed the best probiotic potential. These strains can be used for the preparation of probiotic products. Many probiotics are commercially available in local markets, but their probiotic potential in a local diet is questionable. Moreover, this study provides an avenue to the scientific community for the exploration of LAB through cost-effective means for the development of functional components of dairy products.

## Figures and Tables

**Table 1:** Acid and bile salt tolerance of presumptive LAB isolates after 3 h of incubation.

Isolate	pH Tolerance			Bile Tolerance		
	pH (2.0)	pH (3.0)	pH (7.0)	0.5	1.0	Control
	log CFU/mL			log CFU/mL		
NMCC-1	6.12 ± 0.06	8.61 ± 0.07	8.44 ± 0.06	7.10 ± 0.05	6.06 ± 0.04	8.70 ± 0.03
NMCC-2	6.39 ± 0.07	8.79 ± 0.09	8.56 ± 0.04	7.77 ± 0.03	6.10 ± 0.06	8.80 ± 0.05
NMCC-4	6.09 ± 0.10	8.45 ± 0.05	8.34 ± 0.08	7.50 ± 0.06	6.12 ± 0.10	8.66 ± 0.11
NMCC-6	6.17 ± 0.11	8.44 ± 0.08	8.55 ± 0.07	7.60 ± 0.07	6.07 ± 0.05	8.70 ± 0.04
NMCC-7	6.22 ± 0.05	8.59 ± 0.07	8.34 ± 0.08	7.68 ± 0.05	6.22 ± 0.06	8.540 ± 0.09
NMCC-8	6.11 ± 0.10	8.45 ± 0.05	8.34 ± 0.05	7.45 ± 0.04	6.24 ± 0.03	8.11 ± 0.08
NMCC-10	6.12 ± 0.08	8.43 ± 0.09	8.55 ± 0.06	7.19 ± 0.04	6.35 ± 0.08	8.23 ± 0.09
NMCC-13	6.32 ± 0.04	8.56 ± 0.06	8.34 ± 0.03	7.11 ± 0.07	6.01 ± 0.09	8.32 ± 0.07
NMCC-14	6.40 ± 0.08	8.78 ± 0.04	8.59 ± 0.04	7.60 ± 0.03	6.17 ± 0.05	8.76 ± 0.08
NMCC-15	6.10 ± 0.07	8.43 ± 0.09	8.55 ± 0.06	7.55 ± 0.09	6.11 ± 0.04	8.70 ± 0.04
NMCC-18	6.39 ± 0.05	8.56 ± 0.06	8.34 ± 0.03	7.65 ± 0.06	6.18 ± 0.08	8.70 ± 0.07
NMCC-17	6.40 ± 0.08	8.79 ± 0.08	8.59 ± 0.03	7.87 ± 0.04	6.12 ± 0.04	8.55 ± 0.08
NMCC-27	6.44 ± 0.04	8.77 ± 0.05	8.55 ± 0.06	7.55 ± 0.08	6.19 ± 0.02	8.67 ± 0.04
NMCC-28	6.43 ± 0.06	8.78 ± 0.07	8.51 ± 0.05	7.49 ± 0.04	6.11 ± 0.06	8.50 ± 0.09
NMCC-18	6.31 ± 0.04	8.69 ± 0.08	8.41 ± 0.07	7.60 ± 0.06	6.05 ± 0.08	8.55 ± 0.05

Values (*n* = 3) are mean ± standard deviations.

**Table 2:** Morphological and biochemical characterization of bacterial isolates on MRS agar.

Characteristics	Selective Bacterial Strains			
	NMCC-2	NMCC-14	NMCC-17	NMCC-27
Morphological characterization				
Gram staining	+ve	−ve	+ve	+ve
Shape	Cocci	Rod	Cocci	Rod
Form	Round	Circular	Circular	Circular
Surface	Shiny	Smooth	Smooth	Smooth
Color	Creamish white	Creamy white	Creamy White	Creamy white
Margin	Entire	Undulate	Entire	Entire
Elevation	Raised	Umbonate	Convex	Convex
Opacity	Opaque	Opaque	Opaque	Translucent
Biochemical characterization				
Catalase	−ve	−ve	−ve	−ve
Oxidase	−ve	−ve	−ve	−ve
Indole	−ve	−ve	−ve	−ve
Citrate	+ve	+ve	+ve	+ve
Methyl red	+ve	+ve	−ve	−ve
Triple sugar iron	−ve	−ve	−ve	−ve
Urease	−ve	−ve	−ve	−ve
Gas from Glucose	+ve	+ve	+ve	+ve
Fermentation	Homo	Homo	Homo	Homo

**Table 3:** Hemolytic and bile salt hydrolyzing (BSH) potential of the isolated LAB strains.

NMCC Strain	BSH	Hemolytic Activity	DNAs
NMCC-2	+	Gama	-
NMCC-14	++	Gama	-
NMCC-17	++	Gama	-
NMCC-27	+	Gama	-

**Table 4:** Antibiotic resistance profiles of selected LAB strains.

NMCC Strain	Streptomycin (10 ug)	Ciproflaxin (20 ug)	Vancomycin (30 ug)	Metronidazole (10 ug)	Ampicillin (5 ug)	Chloramphenicol (30 ug)	Kanamycin (30 ug)	Erythromycin (15 ug)	Penicillin (10 ug)	Tetracycline (30 ug)
NMCC-2	R	R	R	R	I	S	R	S	R	R
NMCC-14	R	R	R	R	I	S	R	S	R	R
NMCC-17	R	R	R	R	I	S	R	S	R	R
NMCC-27	R	R	R	R	I	S	R	S	R	R

Streptomycin, ciprofloxacin, vancomycin, metronidazole, ampicillin, chloramphenicol, kanamycin, erythromycin, penicillin, tetracycline, zone of inhibition, (R) Resistant, (I) Intermediate resistance, (S) Susceptible.

**Table 5:** Antibacterial activity of LAB strains against food pathogens and diameter (mm) of the respective inhibition zones.

NMCC Strains	Test Pathogen				
	*E. coli*	*Pseudomonas aeruginosa*	*Staphylococcus aureus*	*Listeria monocytogenes*	*Bacillus* *cereus*
NMCC-2	+	+	++	-	-
NMCC-14	+++	++	+++	+	+
NMCC-17	++	+	++	+	+
NMCC-27	+	-	-	+	+

Zone diameter: -: 0 mm; +: 0–4 mm; ++: 8–12 mm; +++ >12 m ATCC: American type culture collection, Virginia, USA. *E. coli* (ATCC8739); *Pseudomonas aeruginosa* (ATCC9027), *Staphylococcus aureus*; (ATCC6538); *Listeria monocytogenes* (ATCC13932), *Bacillus cereus* (ATCC-11778).

**Table 6:** Fermentation properties of isolated *Lactobacillus fermentum* strains in milk.

Strain	Protein, %	Lactose, %	pH	WHCa, %	Syneresis, g Water/100 g Milk	Dynamic Viscosity, sec
NMCC-14	4.8 ± 0.9 ^b^	4.4 ± 0.10 ^a^	3.7 ± 0.09 ^c^	36 ± 1.55 ^a^	21 ± 1.8 ^b^	6.1 ± 0.07 ^a^
NMCC-17	4.4 ± 0.06 ^a^	4.3 ± 0.08 ^a,b^	3.6 ± 0.05 ^b^	37 ± 4.3 ^b,a^	24 ± 2.8 ^a^	5.6 ± 0.15 ^a,b^
Pre-fermented milk	4.2 ± 0.09 ^d^	4.8 ± 0.07 ^a,d^	6.8 ± 0.9 ^c^	Nil ^d,a^	Nil ^d^	Nil ^d^

Data is measured by 2-sided Tukey’s HSD. Different subscripts lowercase letters ^a–d^ indicate significant differences (*p* < 0.05) within the same columns.

## Data Availability

Requests to access the datasets should be directed to the corresponding author.
